# The proceedings of the Tenth Symposium on Lactic Acid Bacteria

**DOI:** 10.1186/1475-2859-10-S1-S1

**Published:** 2011-08-30

**Authors:** Colin Hill, Michiel Kleerebezem, Jan Kok

**Affiliations:** 1 University College Cork, Microbiology Department, Cork, Ireland; 2 NIZO Food Research BV, 6710 BA EDE, The Netherlands; 3 University of Groningen, Centre for Life Sciences, 9747 AG Groningen, The Netherlands

## 

We were delighted to be asked by Microbial Cell Factories to act as guest editors for the manuscripts in this issue associated with the LAB10 Symposium. The LAB Symposia have been very important benchmarks in the careers of many LAB researchers, charting the development of this field from the very beginnings of our understanding of the molecular biology and physiology of these hugely interesting organisms right through to the current sophisticated ‘omics’ era. While we have significantly improved our understanding of the basic physiology and the key roles of individual organisms and the contributions of individual molecular mechanisms to the many industrial applications of LAB, our recognition and investigation of the potential of these organisms in human health has been one of the most rewarding aspects of the last few decades. The nine meetings to date have served to bring the global LAB community together every three years to review progress and the plan for the future in a friendly, collaborative setting. It is not an exaggeration to suggest that the Symposia have been a key factor in the extraordinary world of LAB biology, one in which direct competition has peacefully co-existed with a genuine warmth and collaborative spirit. We expect and hope that the tenth meeting will continue this tradition of ‘coopetition’.

The papers contained in this issue are linked to oral presentations at LAB10 by some of the leaders and up-and-coming scientists in our field, and we hope that this collection of manuscripts will fulfil at least two roles. Firstly, they will give an opportunity for a wider audience to evaluate progress in the field, and secondly, they will prove invaluable in the future as a record of the state of the art at this point in our on-going engagement with these industrially important microorganisms.

The manuscripts (both reviews and original papers) cover the main thematic areas of the meeting; Evolution, Systems Biology and Metabolic Engineering, Host Microbe Interaction, Nutrition and Health, Preservations and Stress, and fermentation and Application. We have sought to maintain the usual high standard applied to all MCF submissions for the papers in this issue, and so each paper was subjected to the usual peer review process. We thank the MCF Editor-in-Chief Antonio Villaverde and Helen Whitaker of BioMed Central for their support during the editorial process, the authors for their prompt response to our requests for a rapid process and, most of all, the reviewers who gave their time and effort to review the manuscripts.

## Competing interests

The authors declare that they have no competing interests.

